# Immunobiology of Uveal Melanoma: State of the Art and Therapeutic Targets

**DOI:** 10.3389/fonc.2019.01145

**Published:** 2019-11-05

**Authors:** Maria Sofia Basile, Emanuela Mazzon, Paolo Fagone, Antonio Longo, Andrea Russo, Matteo Fallico, Vincenza Bonfiglio, Ferdinando Nicoletti, Teresio Avitabile, Michele Reibaldi

**Affiliations:** ^1^Department of Biomedical and Biotechnological Sciences, University of Catania, Catania, Italy; ^2^IRCCS Centro Neurolesi Bonino Pulejo, C.da Casazza, Messina, Italy; ^3^Department of Ophthalmology, University of Catania, Catania, Italy

**Keywords:** uveal melanoma, inhibitory checkpoints, immunotherapy, immune-escape, immune-privilege

## Abstract

Uveal Melanoma (UM) represents the most common primary intraocular malignant tumor in adults. Although it originates from melanocytes as cutaneous melanoma, it shows significant clinical and biological differences with the latter, including high resistance to immune therapy. Indeed, UM can evade immune surveillance via multiple mechanisms, such as the expression of inhibitory checkpoints (e.g., *PD-L1, CD47, CD200*) and the production of *IDO-1* and soluble *FasL*, among others. More in-depth understanding of these mechanisms will suggest potential targets for the design of novel and more effective management strategies for UM patients.

## Introduction

Uveal melanoma (UM) is a malignant cancer of the eye that is thought to arise from the melanocytes within the uveal tract of the eye. It differs from cutaneous melanoma (CM), which arises from skin melanocytes, and has distinct clinical and biological features. UM, with an annual incidence of six cases per million, is the most common primary intraocular malignant tumor in adults. It mainly originates from the choroid (~85%), while the remaining cases arise from the ciliary body (5–8%) and the iris (3–5%) (1).

Cutaneous and uveal melanocytes have the same embryonic origin and cellular function, however, they undergo different tumoral transformation processes (2). The majority of CMs (~80%) present mutations in *BRAF, NRAS*, and *NF1* genes (2). Instead, in UM, the most common mutations involve *GNAQ/11* (83% of the cases) and recurrent alterations can be found on the *BAP1* gene (~40%) (2). CM shows several cytogenetic alterations, involving loss of chromosomes 4, 5, 6q, 8p, 9p, 10q, 11q, 12q, 14, 15, 16, 21, and 22 (3), and gain of 1q, 6p, 7, 8q, 18, and 20q (4, 5). In UM, chromosomal aberrations mainly include monosomy 3 (50%) and 6p and 8q gain. UM tumors with monosomy 3 and polysomy 8q have high metastatic risk and a poor prognosis (6, 7). Ludmil and collaborators have shown that CM has the highest somatic mutation prevalence (8), while UM has low somatic mutation rates (9). It is believed that a high mutational burden is predictive of the response to immunotherapy (10), as the neoantigens that derive from tumor-specific mutations can be targets for anti-tumor immune responses. Therefore, the reduced number of neoantigens on UM cells may explain why immune-checkpoint inhibitors are insufficient in UM but can be effective in CM. However, as a low mutational load may also bring the activation of neoantigen-specific T cells (11, 12), it is reasonable to believe that the tumor microenvironment and intrinsic cancer cell phenotypic patterns may be pivotal in the regulation of the ability of T cells to respond to cancer-specific antigens.

In this review, we will discuss key aspects of the immunobiology of UM and potential novel immunotherapeutic targets.

## The Eye: An Immune-privileged Site for Uveal Melanoma?

The eye has been proposed to be an immunologically privileged site, possibly providing UM with a protective niche. This protection has been attributed to cell surface molecules and soluble factors able to impair, weaken, or disturb the immune system. The immune privilege of the eye is instrumental to protecting ocular tissues and preserving vision from damage that may occur following inflammatory reactions (13, 14). Both physical and biochemical mechanisms maintains the immune privilege of the eye (13, 15, 16). The intraocular compartments are separated from the blood circulation by the blood-ocular-barrier, which comprises the blood-aqueous barrier and the blood-retinal barrier (15). The blood-aqueous barrier is made up of tight junctions between the endothelial cells of the ciliary blood vessels and between the lining epithelial cells (15). The aqueous humor is a transparent and colorless medium that is present in the anterior and posterior chambers of the eye. The aqueous humor is secreted by the ciliary epithelium and enters the posterior chamber. Afterwards, it flows around the lens and the pupil into the anterior chamber. Finally, the aqueous humor leaves the eye by passive flow at the anterior chamber angle, in the supraciliary and suprachoroidal space, through the choroidal vessels or through scleral pores (17, 18). In the early seminal work by Taylor and colleagues (19), it was found that primed T cells, activated *in vitro* in the presence of the aqueous humor, produced lower levels of IFN-γ and IL-4 with generation of TGF-β-producing regulatory T cells. TGF-β is an immunomodulatory cytokine primarily produced by Th3 cells that exhibits multiple immunosuppressive properties and has been shown to counteract immunoinflammatory and autoimmune responses both *in vitro* and *in vivo* (20, 21). Recent studies have indicated that, through its immunosuppressive properties exerted in the tumor microenvironment, TGF-β may play a pathogenic role in oncogenesis by suppressing anti-cancer cell-mediated immune responses. On this basis, much attention has recently been focused on the possibility that specific inhibitors of TGF-β, such as antibodies, antisense molecules, and small-molecule tyrosine kinase inhibitors, may represent novel therapeutic approaches for the treatment of certain forms of cancers, possibly including UM (22, 23). In addition, apart from being rich in TGF-β, other studies have demonstrated that the aqueous humor contains large amounts of the pleiotropic cytokine Macrophage Migration Inhibitory Factor (MIF), which promotes immune privilege by inhibiting NK cell activity (24), though MIF possesses proinflammatory properties that qualify it as an important mediator of several autoimmune diseases such as multiple sclerosis and Guillain Barrè syndrome (25, 26). Recent data also highlight that MIF can activate multiple oncogenic pathways, including the inhibition of p53, production of HIF-1α (Hypoxia-inducible factor 1-alpha), and activation of the PI3K/Akt/mTOR pathway. These observations have attracted much attention to the role of MIF in the pathogenesis of several types of cancer, including glioblastoma, melanoma, and head and neck cancer, among others, and on the possible use of specific MIF inhibitors in these diseases (27–30).

Other molecules that have been detected in the aqueous humor and could dampen anti-tumor immune responses include α-melanocyte-stimulating hormone (α-MSH), calcitonin gene-related peptide (CGRP), vasoactive intestinal peptide (VIP), and somatostatin, by which delayed-type hypersensitivity reactions are suppressed and Treg cell activity is induced (13, 31, 32).

Finally, iris and ciliary body epithelial cells are able to prevent T cell activation and proliferation via direct cell-to-cell contact (33).

The absence of afferent lymphatics also limits the homing of immune cells to and from the secondary lymphoid organs. However, studies by Camelo and colleagues have shown that, after intracameral and subconjunctival injection, antigens reach the ipsilateral head and neck lymph nodes via the conjunctival lymphatics, and that antigen administration into the anterior chamber is internalized by ocular Antigen Presenting Cells (APCs) and presented in a tolerogenic fashion in the spleen (34, 35). This is referred to as anterior chamber-associated immune deviation (ACAID). In ACAID, eye-derived APCs promote the expansion of tolerogenic B cells in order to induce invariant natural killer T cells and antigen-specific Tregs. In particular, afferent CD4+ Tregs act in the secondary lymphoid organs to suppress the initial activation and differentiation of naïve T cells into Th1 effector cells, while efferent CD8+ Tregs act in the eye, inhibiting the delayed hypersensitivity responses [reviewed by (36)].

Apparently, this condition of immune privilege should promote the incidence of intraocular tumors; however, as reported in the American Cancer Society 2008 statistics, UM is about 15 times less frequent than CM. Despite this, it is likely the UM may receive an advantage from the ocular immune privilege that, coupled to the acquisition of immune-regulatory properties, could eventually result in clinically relevant tumors.

After leaving the eye, the ability of UM cells to express pro-oncogenic molecules such as indoleamine dioxygenase-1 (*IDO-1*), *MIF*, and *PD-L1* (37–39) re-establish their immune privilege and provide the possibility to set up metastatic disease.

Also, in contradiction with the immune surveillance hypothesis, in the case of UM, the immune system seems to promote cancer development, maintenance, and progression. Indeed, the presence of Tumor-Associated Lymphocytes (TILs) and Tumor-Associated Macrophages (TAMs) correlates with a poor prognosis (40–42).

It is of note that the choroid is located outside of the above-mentioned outer blood-retina barrier. Choroidal capillaries are fenestrated and very leaky (43, 44); therefore, the choroidal space is considered to be exposed to the systemic immune surveillance. It is possible that, once the primary choroidal melanoma grows and breaks the outer blood-retina-barrier, the tumor could utilize the immune suppressive mechanisms of the affected eye to tolerize the immune attacks against melanoma cells.

## Immunobiology of Primary UM

UM cells express tumor-specific antigens, including the Melanoma Antigen Gene (MAGE) family proteins, premelanosome protein gp100, and tyrosinase (45–47), that are recognized by elements of the immune system. Accordingly, *in vitro* data show that circulating CD8+ CTL from UM patients or from primary UMs are able to lyse UM cells (48–50). NK cells are able to induce cytotoxicity in UM cell lines, such as OCM-3 (32). However, both the innate and adaptive effector immune responses can be circumvented by UM cells. Some of these strategies are common to those that provide the immune privilege to the eye. Indeed, the immune privilege is not absolute nor permanent, and it can be overcome as is shown by the development of uveitis and the rejection of corneal transplant.

On the other hand, preclinical data have demonstrated that the intraocular transplantation of ultraviolet light (UV)-induced tumors in syngeneic mice subjected to CTL-mediated rejection and that the adoptive transfer of CD8+ TILs in immune-deficient mice challenged with intraocular UV-induced tumors exhibited anti-cancer actions (51). Altogether, these data provide evidence that UM cells put in place specific immune escape mechanisms responsible for its progressive course and bad prognosis.

### Resistance to Cell-Mediated Immune Responses

Natural killer (NK) cells have been shown to control the growth of liver metastases (52). Decreased tumor expression of Class I MHC molecules, ligands for NK inhibitor receptors, is associated with longer metastasis-free survival (53), while the loss of NK activator receptors (i.e., MIC-A and MIC-B) is associated with tumor progression (54). Cytotoxic T lymphocytes (CTL) and Natural Killer (NK) cells exert anti-tumor functions by inducing apoptosis via the activation of the death receptors of the TNF superfamily, including TNF-α, TRAIL, and FasL. However, UM cells are resistant to FasL-induced apoptosis (55). Indeed, the production of a soluble form of FasL from UM cells protects UM cells from apoptosis as, by acting in an autocrine manner, it binds Fas expressed by UM cells themselves, blocking the engagement of Fas expressed on CTL and NK cells, which, given its trimeric structure, is more than 1,000 times more efficient in inducing apoptosis (55).

Moreover, as previously stated, the aqueous humor contains TGF-β and MIF, which have profound inhibitory effects on NK cells (24). In particular, TGF-β and MIF act sequentially to dampen NK function as MIF provides immediate inhibition (24), while TGF-β exerts long-term inhibitory function (56).

The presence of TILs and TAMs in UMs correlates with a poor prognosis (40–42). Although this observation is unexpected and contrasts with data from other cancer types, there may be multiple reasons for this peculiar UM feature. One possible explanation is that the production of pro-inflammatory cytokines, and in particular IFN-γ, is able to induce the upregulation of MHC class I molecules, which help UM cells to escape from NK cytolysis and promote the expression of IDO-1 and of inhibitory immune checkpoints, e.g., PD-L1. Another and not mutually exclusive explanation is that the IL-2 secreted by infiltrating lymphocytes may have a proliferative effect on UM cells as well. It has been found that UM cells express the receptor for IL-2 and IL-15, which may promote their survival and growth and deplete essential factors for the action and proliferation of both T and NK cells (57). Moreover, IFN-γ can sustain cancer growth by inducing a downregulation of tumor antigens (58, 59).

However, the mechanisms regulating the cancer-microenvironment crosstalk remain elusive. In a recent study by Rothermel et al. (60), analysis of TILs cultures from cutaneous and UM showed that UM TILs were predominantly CD4+, while in CM were mainly composed of CD8+ T cells. Also, the absence of melanin pigmentation in the primary tumor was strongly correlated with highly reactive UM TILs. It is believed that UM cells interact with infiltrating cells and skew their phenotype to an immune-regulatory type. Recent studies have identified the presence of CD4+ and Forkhead box P3 (FoxP3)+ Treg cells within primary UMs, and their frequency has been found to correlate with metastatic dissemination (61, 62). In patients with primary UM, while circulating anti-tumor CD3–CD56^dim^ NK cells and CD8+ and double-negative CD3+CD56+ NK-T cells decrease, pro-tumoral ICOS+CD4+FoxP3+ Treg cells increase (63), further supporting a role for Treg in tumor progression. The striking correlation between tumor size and high metastatic risk primary UMs infiltrated by CD8+ T cells seems to suggest that UM may promote the generation of CD8+ Tregs (41, 64). Accordingly, Streilein and Niederkorn showed that elimination of CD8+ Treg in a murine model of UM was sufficient to induce tumor rejection (65). It has also been found that patients with primary UMs and liver metastases bear increased frequencies of circulating CD11b+CD15+ cells, which could represent immunosuppressive myeloid-derived suppressor cells (63, 66). Interestingly, untreated metastatic UM (and breast cancer patients, as well) have an increased percentage of circulating CD127–CD25–CD4+ T cells in the blood, as compared to healthy people. This cell population, considered to be “chronically stimulated” CD4+ T cells, shares features observed in anergic cells from tumor-bearing mice, i.e., reduced proliferation ability and diminished cytokine production. Accordingly, these cells have significant transcriptome overlapping that mirrors that of mouse anergic cells (67).

An increased body of data is accumulating for *IDO-1* as an evading mechanism put in place by cancer to elude the immune surveillance (68). A potential role for *IDO-1* has already been described in several tumor types, including colorectal cancer (69), hepatocarcinoma (70), endometrial cancer (71), and CM (72). T lymphocytes require the amino acid tryptophan for survival and clonal expansion. The enzyme IDO-1 catalyzes the rate-limiting step in tryptophan catabolism, which leads to the oxidation of L-tryptophan to N-formylkynurenine. *IDO-1* is expressed by the retina, iris/ciliary body, lens, and cornea (73, 74). Although, Chen and colleagues failed to observe the expression of IDO-1 in both primary UM samples and in liver UM metastases (75), UM cell lines exposed *in vitro* to IFN-γ, significantly upregulate *IDO-1* expression (75). These data suggest a potential role for *IDO-1* as an immune escape mechanism. Despite these data, the role of *IDO-1* in metastatic UM remains questionable, as specific anti-IDO-1 strategies have yet to prove efficacy in UM patients.

Closely related to *IDO-1*, tryptophan 2,3-dioxygenase (TDO) is a heme-containing enzyme, encoded by the *TDO2* gene. Terai et al. (76) have recently reported that *TDO2* mRNA is expressed by 62% of primary UM and correlates with a poor prognosis. Also, the Authors show that TDO expression is upregulated by 3.5-fold upon *in vitro* stimulation of UM cells with recombinant TNF-alpha. These observations point to a complementary and, possibly, overlapping role of TDO and IDO-1 in the immune-evading strategies of advanced UM, and, therefore, novel pharmacological interventions aimed at inhibiting the kynurenine pathway, targeting both enzymes simultaneously, are strongly warranted.

### Inhibitory Immune Checkpoints in Primary Uveal Melanoma

The immune system uses a diverse set of antigens to distinguish tumor cells from their healthy cells. The amplitude of the T cell response is regulated by both co-stimulatory and inhibitory molecules, known as “immune checkpoints,” which are essential for the maintenance of self-tolerance. In cancer, multiple inhibitory checkpoints may be modulated, including programmed death ligand-1/2 (PD-L1/2), CD47, Galectin 9, and TNFRSF6B, for which ligands expressed on T cells or APCs may act synchronously or sequentially to promote overall suppression of the immune responses (77). Robertson et al. (78), by performing a multiplatform analysis of 80 primary UM samples from the TGCA dataset, identified four distinct UM subtypes, two with poor prognosis monosomy of chromosome 3 (named M3) and two with better prognosis disomy of chromosome 3 (named D3). Deconvolution analysis of both DNA methylation and RNA-seq data revealed that a CD8+ T cell infiltrate was present in ~30% of M3 samples, whereas it was almost absent in D3 samples. Also, they found that genes co-expressed with CD8A were associated with immunosuppression (*IDO1, TIGIT, IL6, IL10, and FOXP3*), T cell migration (*CXCL9* and *CXCL13*), cell-mediated cytotoxicity (*PRF1* and *GZMA*), and interferon-γ signaling (*IFNG, IFNGR1, and IRF1*). Moreover, *HLA* expression was higher in M3 samples as compared to D3 samples and correlated with *CD8A* expression (78). Accordingly, Maat and colleagues, in a comparative immunohistochemical analysis of M3 and D3 samples, observed that M3 tumors have a significantly higher number of infiltrating macrophages and express higher levels of MHC class I and II (79).

Elucidation of the complex network of stimulatory and inhibitory signals that contribute to immune regulation and its dysregulation in cancer may lead to more effective therapeutic opportunities to enhance anti-tumor immune responses.

#### PD-1/PD-L1

PD-1 is expressed on T lymphocytes and has the two ligands, PD-L1 (also known as B7-H1 or CD274) and PD-L2, that belong to the B7 superfamily. Both are expressed on APCs and cancer cells. A lack of expression of PD-L1 has been observed in primary UM. Yang and collaborators (80) found that PD-L1 was not expressed by primary UM *in situ*, and, similarly, Kaunitz et al. (81) observed that only 10% of UM samples expressed PD-L1. Interestingly, when present, the expression of PD-L1 on tumor cells was mainly associated with the presence of CD8+ T-lymphocytes, consistent with an adaptive mechanism of expression. This is in line with the observation that, in UM cell lines, derived from primary tumors *PD-L1* and *PD-L2*, expression significantly increased under inflammatory conditions. Also, *PD-L1* was found to inversely correlate with OS, PFS, and thickness of the tumor (82).

#### CD47

CD47 is an immunoglobulin-like domain containing protein expressed by the tumor cell surface that inhibits macrophage phagocytosis by binding the signal regulatory protein α (SIRPα) on APCs. CD47 downregulation is associated with macrophage phagocytosis of senescent or damaged cells. On the contrary, upregulation of CD47 inhibits phagocytosis. The interaction between CD47 and SIRPα activates the tyrosine phosphorylation of the cytoplasmic region of SIRPα, thus recruiting the tyrosine phosphatase, SHP-2, which acts by dephosphorylating its substrates, and functions as a negative signaling regulator. CD47 is overexpressed in many different cancer cell types and represents an independent negative prognostic factor (83, 84). We have shown that UM cells lines dramatically upregulate *CD47* expression after incubation with an activated T cell supernatant, and that higher levels of *CD47* were associated with significantly lower disease-free survival time. Accordingly, the expression of *CD47* in primary UM samples was an independent predictor of recurrence disease (82). In UM, we also found that *CD47* levels did not significantly change in the different stages of the disease, and that patients with the lowest expression of *CD47* had improved progression-free survival (PFS), even after correcting for the presence of *BAP1, GNAQ*, and *GNA11* mutations (85). Interestingly, deconvolution analysis of infiltrating immune cell populations showed a significantly higher proportion of CD4+ and CD8+ T cells in patients with high CD47 levels, with the most represented populations being the Th2, Treg, and CD8+ TCM cells (85). Finally, we demonstrated that a large number of transcripts are differentially expressed between tumors expressing high and low levels of *CD47*, with a significant enrichment of interferon IFN-alpha regulated genes (85).

#### CD200

CD200 (also known as OX-2) is a type 1a glycoprotein, capable of modulating the immune system via its inhibitory receptor CD200R, which is expressed on both myeloid and lymphoid cells. It contains two extracellular immunoglobulin domains and a small intracellular domain with no known signaling motif. CD200R expression, the cognate ligand for CD200, is mainly restricted to the myeloid lineage of cells (86). Accordingly, *CD200*-deficient mice show hyperactivation of macrophages and enhanced inflammation in autoimmune disease models (87). *CD200* has been found to be a good predictor of recurrent disease in UM (82).

#### Gal9 and TNFRSF6B

A potential prognostic role for *GAL9* and *TNFRSF6B* has also been recently evaluated (82). Higher levels of expression of these proteins have been associated with a better PFS. Galectin 9 is protein encoded by the gene GAL9 that, interacting with its cognate receptor, TIM-3, is able to inhibit Th1 responses, triggering the apoptosis of CTLs and increasing Tregs suppressive activity. Conversely, it was shown in a preclinical model of melanoma that GAL9 increased the NK-mediated cytolysis of cancer cells. Accordingly, a recent meta-analysis on solid cancer patients showed that higher levels of GAL9 correlated with improved OS, reduced depth of invasion, and negative distal tumor dissemination (88).

TNFRSF6B belongs to the tumor necrosis factor receptor superfamily and functions as a decoy receptor for FasL, tumor necrosis factor-like ligand 1A (TL1A), and lymphotoxin analogs (LIGHT). *TNFRSF6B* expression correlated with reduced OS in patients with solid tumors, but it did not influence recurrence-free survival (89). Along the same lines, higher levels of *TNFRSF6B* were associated with longer PFS in UM (82).

#### Nitric Oxide

Nitric oxide (NO) is an endogenous gas produced from neural, constitutive, or inducible nitric oxide synthases (NOS) from L-arginine. Together with Hydrogen Sulfide and Carbon Monoxide, NO represents the main gaseous endogenous system in the body. It is of interest that recent data indicate that these gas-signaling molecules play critical roles in regulating signal transduction and cellular homeostasis. Interestingly, through various administrations, these molecules also exhibit potential in cancer treatment (90, 91). As, out of the three gases, the role of NO in cancer and UM is the most widely known, we will briefly review the literature of NO in UM. NO plays pleiotropic biological functions ranging from blood pressure homoeostasis to the regulation of responses to infectious agents and modulation of immune responses and oncogenesis. Depending on the concentration and location of the effects, NO may often exert dichotomic roles in the regulation of the same process (91). In the setting of cancer, depending on the type of tumor and doses and location of its action, NO has been shown to exert both anti- and pro-oncogenic properties (92, 93). As a matter of fact, expression of iNOS has been shown to represent a negative prognostic factors for multiple types of cancer, including primary UM (94, 95). In particular, recent evidence seems to indicate that NO may act as an addition local immune checkpoint inhibitor, favoring immune evasion of the tumor, by modulation of the acquisition of stem cell-like capacities, the metabolic reprogramming of tumor-infiltrating immune cells, and the induction of myeloid-derived suppressor cells that deplete arginine, via the iNOS pathway, and consequently inhibit T cell function (96, 97).

However, despite these above-mentioned data that strongly support the concept that endogenous NO represents a powerful oncogenic mediator in the maintenance and progression of UM, data by ourselves and others indicate that exogenous NO-derivatives of parental drugs possess enhanced anticancer properties in preclinical models of blood cancer, bladder and prostate cancer, and cutaneous melanoma (98–102).

Dual strategies, therefore, could be envisaged aimed at targeting the NO-producing enzymes and the signaling pathway mediated by NO in UM. Further studies are needed to highlight this concept, along with an evaluation of the other endogenous gases and their donors, H_2_S in particular, in UM.

### Immunobiology of UM Liver Metastasis

The principal organ for UM metastasis is the liver, which is involved in up to 87% of patients with metastatic disease. The liver is often the first metastatic site in UM, and in almost 40% of patients it is the only site of systemic metastasis. Unlike CM, where metastasis to the central nervous system (CNS) occurs in 40–60% of cases, only 4–15% of UM spread to the CNS. Holfort et al. (103) found that UM patients with CNS metastasis either had multiple organ metastasis, which included the CNS, or showed selective CNS metastasis, and, interestingly, a longer interval from primary tumor to CNS metastasis was observed in patients with selective CNS metastasis as compared with the multiple organ metastatic group (103). The peculiar metastatic pattern of UM cannot be explained only by circulation, as the lungs are the first organ that UM cells encounter during their hematogenous spreading. Other factors should therefore be involved, although the exact mechanisms that guide the establishment of liver metastasis in UM remain speculative.

It is believed that the homing of UM cells to the liver is dependent on the expression of the CXCR4, the chemokine receptor for CXCL12, which is highly expressed in the liver (104). Recent data have also demonstrated that exosomes from UM cells expressing integrin α_v_β_5_ are taken up by liver cells, inducing the establishment of a pre-metastatic niche that promote liver tropism (105, 106). However, it is likely that the immunological microenvironment of the liver may favor UM metastatic growth, protecting cancer cells from cytotoxic immune responses [reviewed by (107)]. The Liver must be considered an immuno-modulatory organ as it is continually exposed to exogenous antigens, such as food allergens and low levels of lipopolysaccharide, from the gut. The peculiar anatomy of the liver promotes both direct and indirect priming of lymphocytes, and it can modulate the immune response to pathogens and tumor cells through its ability to induce antigen-specific tolerance. Several highly specialized cellular types are located within the sinusoidal structure and in the parenchyma of the liver, including liver sinusoidal endothelial cells (LSECs), Kupffer cells (KCs), NK cells, and NKT cells. LSECs are capable of receptor-mediated phagocytosis and can present blood-derived antigens to both CD4+ T and CD8+ T cells. Upon stimulation, LSECs also produce the chemokines, CXCL9 and CXCL10, that recruit T lymphocytes. On the other hand, LSECs may express the inhibitory immune checkpoint PD-L1, thus controlling T cell activation (108–112).

KCs, the most abundant tissue macrophages in the body, reside within the sinusoidal vascular space and are able to recognize microorganisms and tumor cells via the C-type lectin receptor Dectin-2 (113). However, KCs may also produce soluble factors, such as IL-10 and prostaglandin E2 (PGE2), that induce a downregulation of MHC class II expression and of the costimulatory molecules, CD80 and CD86, on LSECs, dampening antigen presentation to Helper T cells (114).

The liver also hosts diverse populations of both resident and transiting lymphocytes that are strikingly different from those observed in other tissues and in the circulation. Approximately half of the population of hepatic lymphocytes are represented by NK cells. Liver-resident NK cells are compose of CD49+ NK cells and Eomes^hi^ NK cells, the latter located in the sinusoidal space and accounting for 50% of human liver NK cells (115). NK cells respond to a variety of cell-surface ligands expressed by damaged, tumoral, or infected cells, and exert direct cytotoxicity by releasing cytotoxic granules containing perforin and granzymes (116). NKT cells represent an important immunomodulatory population of the liver. These cells have a restricted TCR repertoire and are able to respond to lipid antigens. However, NKT cells may sustain both inflammatory and anti-inflammatory responses, producing cytokines, such as IFN-γ, IL-4, and IL-17, based on the type of the activating signal (116). NK cells are thought to control metastases growth of UM (117), while NK T cells are able to suppress the cytotoxicity of NK cells via bone marrow-derived cells (118).

Despite the increasing understanding of the immune-phenotypic architecture of the liver, the immune suppressive pathways involved in metastatic UM and the liver tumor microenvironment remain largely elusive. Krishna et al. (119) have recently characterized the immune cell infiltrates in liver metastatic UM and found that CD4+ TILs were located within the tumor, whereas CD8+ TILs tended to be peritumoral. Also, CD68+ and CD163+ TAMs of “indeterminate” morphology were observed, suggesting the presence of protumorigenic M2 macrophages (119). It is worth noting that a meta-analysis of the transcriptomic features of metastatic UM samples (120) found no differences in the expression of genes involved in immune evasion (including HLA molecules, immune checkpoints, cytokines, and anti-inflammatory factors). Hence, we may speculate that intrinsic transcriptomic features of UM cells allow the development and progression of hepatic metastatic disease.

Considering the pre-existing immune tolerance against UM cells, the low mutational burden, and the hepatic immune-modulating microenvironment, it is reasonable that the combination of these factors may promote the more frequent establishment of metastasis in the liver as compared to other organs.

## Immunotherapy in Advanced/Metastatic Uveal Melanoma

Cancer immunotherapy differs from conventional chemotherapeutic agents in that it enhances the immune responses toward tumor cells rather than affecting cancer cell survival and proliferation via radio- or chemical-induced toxicity. Immunotherapy encompasses several subtypes of treatment modality, including vaccination, cell-based therapies using patients' immune cells, and immunomodulatory agents, among which anti-checkpoint inhibitor therapies have been successful in some solid tumors. A list of the current immunotherapy trials enlisted in ClinicalTrials.gov is presented in Table 1.

**Table 1:** Clinical trials on immunotherapy in uveal melanoma.

**Title**	**Status**	**Intervention**	**NCT Number**
**IMMUNE CHECKPOINT INHIBITOR-BASED INTERVENTIONS**			
Yttrium90, ipilimumab, and nivolumab for uveal melanoma with liver metastases	Recruiting	SIR-Spheres® yttrium 90; ipilimumab; nivolumab	NCT02913417
Study of immunotherapy plus ADI-PEG 20 for the treatment of advanced uveal melanoma	Recruiting	ADI PEG20; nivolumab; ipilimumab	NCT03922880
Study of AntiCTLA4 in patients with unresectable or metastatic uveal melanoma	Completed	CP-675,206	NCT01034787
Nivolumab and ipilimumab in treating patients with metastatic uveal melanoma	Active, not recruiting	Ipilimumab; nivolumab	NCT01585194
Trial of nivolumab in combination with ipilimumab in subjects with previously untreated metastatic uveal melanoma	Active, not recruiting	ipilimumab; nivolumab	NCT02626962
Efficacy study of pembrolizumab with entinostat to treat metastatic melanoma of the eye	Active, not recruiting	Pembrolizumab; etinostat	NCT02697630
CAVATAK® and ipilimumab in uveal melanoma metastatic to the liver (VLA-024 CLEVER)	Completed	CVA21; ipilimumab	NCT03408587
Pembrolizumab in treating patients with advanced uveal melanoma	Active, not recruiting	Pembrolizumab	NCT02359851
Ipilimumab and nivolumab with immunoembolization in treating participants with metastatic uveal melanoma in the liver	Recruiting	Ipilimumab; nivoleumab; embolization therapy	NCT03472586
Nivolumab with or without ipilimumab or relatlimab before surgery in treating patients with stage IIIB–IV melanoma that can be removed by surgery	Recruiting	Ipilimumab; nivolumab; relatlimab; therapeutic conventional surgery	NCT02519322
Radioembolization and ipilimumab in treating patients with uveal melanoma with liver metastases	Terminated	Ipilimumab; yttrium Y 90 glass microspheres	NCT01730157
Intravenous and intrathecal nivolumab in treating patients with leptomeningeal disease	Recruiting	Nivolumab	NCT03025256
A study of XmAb®23104 in subjects with selected advanced solid tumors (DUET-3)	Recruiting	XmAb®23104	NCT03752398
A safety and tolerability study of INCAGN02385 in select advanced malignancies	Recruiting	INCAGN02385	NCT03538028
A safety and tolerability study of INCAGN02390 in select advanced malignancies	Recruiting	INCAGN02390	NCT03652077
Nab-paclitaxel and bevacizumab or ipilimumab as first-line therapy in treating patients with stage IV melanoma that cannot be removed by surgery	Active, not recruiting	Bevacizumab; ipilimumab; nab-paclitaxel	NCT02158520
Glembatumumab vedotin, nivolumab, and ipilimumab in treating patients with advanced metastatic solid tumors that cannot be removed by surgery	Withdrawn	Glembatumumab vedotin; ipilimumab; nivolumab	NCT03326258
**CELL-BASED INTERVENTIONS**			
Messenger ribonucleic acid (mRNA) transfected dendritic cell vaccination in high risk uveal melanoma patients	Terminated	Autologous dendritic cells electroporated with mRNA	NCT00929019
Dendritic cells plus autologous tumor RNA in uveal melanoma	Recruiting	Autologous dendritic cells loaded with autologous tumor RNA	NCT01983748
Immunotherapy using tumor infiltrating lymphocytes for patients with metastatic ocular melanoma	Terminated	Aldesleukin; cyclophosphamide; fludarabine; young tumor infiltrating lymphocytes (TIL)	NCT01814046
Adoptive transfer of tumor infiltrating lymphocytes for metastatic uveal melanoma	Recruiting	Tumor infiltrating lymphocytes (TIL)	NCT03467516
Dendritic cell vaccination during lymphoid reconstruction	Completed	Autologous dendritic cells (DC); fludarabine; autologous lymphocyte infusion (ALI)	NCT00313508
**CYTOKINE-BASED INTERVENTIONS**			
PEG-interferon Alfa-2b and thalidomide in treating patients with recurrent or metastatic melanoma	Completed	PEG-interferon alfa-2b; thalidomide	NCT00238329
Dacarbazine and recombinant interferon Alfa-2b in treating patients with primary uveal melanoma with genetic imbalance	Completed	Recombinant interferon alfa-2b; dacarbazine	NCT01100528
Temozolomide and interferon alfa in treating patients with stage III or stage IV melanoma	Completed	Pegylated interferon alfa; temozolomide	NCT00027742
Interferon beta in treating patients with metastatic cutaneous melanoma or ocular melanoma	Completed	Leuprolide; GP100: 209-217(210M) peptide; MAGE-3 peptide; recombinant interferon beta; recombinant human thrombopoietin; etoposide; ifosfamide; G-CSF	NCT00254397
**VACCINE-BASED INTERVENTIONS**			
Vaccine therapy in treating patients with stage III or stage IV melanoma that cannot be removed by surgery	Completed	Incomplete Freund's adjuvant; multi-epitope melanoma peptide vaccine; sargramostim	NCT00089206
Safety and activity of controllable PRAME-TCR therapy in previously treated AML/MDS or metastatic uveal melanoma	Active, not recruiting	BPX-701; rimiducid	NCT02743611
Vaccine therapy in treating patients with melanoma of the eye	Terminated	MART-1 antigen; NA17-A antigen; gp100 antigen; tyrosinase peptide	NCT00036816
Safety and immunogenicity of a melanoma DNA vaccine delivered by electroporation	Completed	Xenogeneic tyrosinase DNA vaccine	NCT00471133
Vaccine therapy in treating patients with metastatic melanoma	Completed	MART-1 antigen; gp100:209-217(210M) peptide vaccine; tyrosinase peptide	NCT00334776
Vaccine therapy in treating patients with stage IIB, stage IIC, stage III, or stage IV melanoma	Completed	Mouse gp100 plasmid DNA vaccine	NCT00398073
Vaccine therapy in treating patients with advanced melanoma	Completed	Incomplete Freund's adjuvant; multi-epitope melanoma peptide vaccine; tetanus toxoid helper peptide	NCT00705640
Vaccine therapy in treating patients with stage III or stage IV melanoma	Terminated	MART-1 antigen; gp100 antigen; incomplete Freund's adjuvant; progenipoietin; tyrosinase peptide	NCT00005841
Vaccine therapy in treating patients with stage IIC-IV melanoma	Completed	gp100 antigen; tyrosinase peptide; recombinant MAGE-3.1 antigen; multi-epitope melanoma peptide vaccine; incomplete Freund's adjuvant; montanide ISA 51 VG; agatolimod sodium	NCT00085189
**COMBINED IMMUNOTHERAPY INTERVENTIONS**			
Autologous CD8+ SLC45A2-specific T lymphocytes with cyclophosphamide, aldesleukin, and ipilimumab in treating participants with metastatic uveal melanoma	Recruiting	Aldesleukin; autologous CD8+ SLC45A2-specific T lymphocytes; cyclophosphamide; ipilimumab	NCT03068624
Safety and efficacy of IMCgp100 vs. investigator choice in advanced uveal melanoma	Recruiting	IMCgp100; dacarbazine; ipilimumab; pembrolizumab	NCT03070392
Vaccine therapy and monoclonal antibody therapy in treating patients with stage IV melanoma	Completed	gp100 antigen; incomplete Freund's adjuvant; ipilimumab	NCT00032045
Monoclonal antibody therapy and interleukin-2 in treating patients with metastatic melanoma	Completed	Aldesleukin; ipilimumab	NCT00058279
Trial of radiation and gene therapy before nivolumab for metastatic non-small cell lung carcinoma and uveal melanoma	Recruiting	ADV/HSV-tk; valacyclovir; SBRT; nivolumab	NCT02831933
Epacadostat and vaccine therapy in treating patients with stage III–IV melanoma	Completed	Epacadostat; MELITAC 12.1 peptide vaccine	NCT01961115
Monoclonal antibody therapy and vaccine therapy in treating patients with resected stage III or stage IV melanoma	Completed	Ipilimumab; Tyrosinase/gp100/MART-1 peptides	NCT00084656
Vaccine therapy with or without interleukin-12 in treating patients with stage III or stage IV melanoma	Completed	gp100 antigen; incomplete Freund's adjuvant; recombinant interleukin-12; tyrosinase peptide	NCT00003339
Vaccine therapy in treating patients with recurrent stage III or stage IV melanoma that cannot be removed by surgery	Completed	Peptide vaccine; GM-CSF; PF3512676	NCT00471471
Monoclonal antibody and vaccine therapy in treating patients with stage III or stage IV melanoma that has been removed during surgery	Completed	MART-1 antigen; gp100 antigen; incomplete Freund's adjuvant; ipilimumab; tyrosinase peptide; adjuvant therapy	NCT00025181
Vaccine therapy and interleukin-12 with either alum or sargramostim after surgery in treating patients with melanoma	Completed	MART-1 antigen; gp100 antigen; incomplete Freund's adjuvant; recombinant interleukin-12; sargramostim; alum adjuvanttyrosinase peptide; adjuvant therapy	NCT00031733
Sargramostim, vaccine therapy, or sargramostim and vaccine therapy in preventing disease recurrence in patients with melanoma that has been removed by surgery	Completed	Sargramostim; tyrosinase peptide	NCT01989572

The immune-based therapies that have improved the overall survival (OS) of CM patients have not yet led to significant clinical benefits in unresectable/metastatic UM patients (121).

For example, while the immunomodulatory antibodies against the antigen associated with cytotoxic T lymphocytes 4 (CTLA-4) and PD-1/PD-L1 have significantly ameliorated the course of metastatic CM, they have failed when translated to UM patients (121). A multicenter retrospective study on UM patients treated with anti-CTLA-4 or anti-PD-1 mAbs revealed that the adjusted OS of patients with immunotherapy was not significantly different from that of patients treated with chemotherapy, with an unadjusted median OS of 13.38 and 11.02 months, respectively (122). Despite this, the increasing understanding of the immunology of cancer may in the future suggest the possibility of novel pharmacological strategies. Since both CM and UM originate from the melanocyte as same precursor, there might be subsequent factors of differentiation or local factors that are responsible for the different responses to immunomodulatory approaches. On the other hand, the first prospective study of ipilimumab in high-risk primary UM in an adjuvant setting showed that DMFS at 36 months was 80%, as compared to a historical DMFS of 50%; in two of 10 patients, however, treatment discontinuation was required due to grade 3–4 toxicity (123).

The PD-1/PD-L1 pathway is responsible for inhibiting T cell activation in the periphery. To date, the largest clinical trial using anti-PD-1 receptor monoclonal antibodies was conducted on 58 metastatic UM patients treated with either pembrolizumab, nivolumab, or atezolizumab. Of the 56 evaluable patients, only 3.6% obtained partial responses and 8.9% presented a stable disease (124). Also, in a prospective observational cohort single-arm study investigating the efficacy and safety of pembrolizumab as first-line therapy for metastatic UM patients, Rossi et al. found that the efficacy of pembrolizumab does not seem particularly different when compared to other agents for metastatic UM, although responding patients had a remarkable disease control (125). This is in sharp contrast to the response observed in patients with CM, where pembrolizumab significantly increased recurrence-free survival as compared to placebo (75.4% vs. 61.0% for the 1-year rate of recurrence-free survival, respectively) (126).

Prospective studies on anti-PD-1 therapy, alone or in combination with other agents, are currently ongoing (127). Disappointing results have also been obtained with ipilimumab, the anti-CTLA-4 antibody. In a retrospective study on 82 Stage 4 UM patients who received ipilimumab, only 5% had an objective response and 29% had stable disease exceeding 3 months. Median OS was 6.0 months and median PFS was 3.6 months, with a 31 and 11% 1-year OS and PFS, respectively (128). Again, this is in strong contrast with data from patients with stage III CM, where ipilimumab was associated with a 5-year rate of recurrence-free survival of 40.8%, as compared with 30.3% in the placebo group, and to a rate of OS at 5 years of 65.4%, as compared with 54.4% in the placebo group (129).

Tremelimumab, an anti-CTLA-4 antibody, has also been tested in a Phase II study on 11 advanced UM patients who had not previously received other immunotherapy drugs. None of them showed clinical benefit (130).

In a Phase II multicenter single-arm open-label study of nivolumab in combination with ipilimumab (NIVO+IPI) in untreated patients with metastatic UM (Clinical trial identification EudraCT:201500442915), ORR was 12%, with disease stabilization in 52% of patients and a Disease Control Rate of 64% (95% CI 50.7–77.3). With a median follow-up of 7.06 months, PFS was 3.27 months and the median OS was 12.7 months, showing that the combination of NIVO+IPI is a feasible option for UM patients (131). Also, in another single-center trial, sequential/concomitant immune-checkpoint inhibitor treatment produced a longer median OS than single-agent ipilimumab or anti-PD1, with a median OS of 23.7 months (sequential ipilimumab and anti-PD-1) vs. single-agent ipilimumab (13.8 months) and single-agent PD-1 (14.7 months) (132).

Accordingly, in a retrospective case series of eight patients treated with ipilimumab and nivolumab combination along with transarterial chemoembolization (TACE) followed by nivolumab maintenance and monthly TACE procedures, two patients showed a partial response, four had stable disease, and the remaining two patient had disease progression (133). Along the same lines, in a preliminary retrospective case series using Yttrium-90 transarterial radioembolization (TARE) and immunotherapy (either ipilimumab, nivolumab, or pembrolizumab) for UM hepatic metastases, it was found that TARE in addition to immunotherapy is safe and effective (134).

TILs treatment has given promising results in metastatic UM, but no definite results have been yet achieved. In a Phase II clinical trial on 21 patients treated with TILs therapy (NCT01814046), seven of 20 patients showed objective tumor regression. On the other hand, when fewer than 3% of tumor-reactive T cells, fewer than 2 × 10^9^ tumor-reactive T cells, or low tumor-induced IFN-γ release were observed, patients underwent poor clinical responses (135). This study suggests that adoptive transfer of TILs with threshold production of IFN-gamma could promote objective tumor regression, but more effort is needed to increase the percentage of responding patients.

Another promising immunotherapeutic approach is represented by the use of the bispecific antibody IMCgp100. IMCgp100 binds the melanocyte protein gp100 on one end and is constituted by an anti-CD3 single-chain variable fragment on the other end. Therefore, IMCgp100 is able to recognize melanoma cells and contextually activate T cells responses, leading to tumor cytolysis. In two Phase I trials, i.e., NCT01211262 and NCT2570308, IMCgp100 treatment was associated with prolonged disease stabilization with a 1-year OS of 73%. Interestingly, IMCgp100 treatment induced an increase in the percentage of infiltrating PD-1+/CD8+ lymphocytes and an upregulation in PD-L1 expression, suggesting the utility of a combinatorial/sequential treatment regime with immune checkpoint inhibitors (136).

The potential therapeutic value of current available immunotherapeutics will be dissected in the near future, following the results of the several ongoing clinical trials. However, the relatively low number of patients with UM and the extremely aggressive nature of this cancer hinders the possibility of easily deciphering the actual potential of immune-based therapies. Also, we should avoid the misconception that the failure of a target-specific approach is synonymous with absence of biological relevance of the selected targets. The failure of a treatment may be due to intrinsic properties of the drug used (e.g., issues with its pharmacokinetics and pharmacodynamics) as well as to the presence of overlapping and/or redundant pathways that may function as a compensatory mechanism to allow tumor growth and progression, hence the need for combining pre-existing therapies. The potential advantages of a combinatory treatment are 2-fold. On one hand, it may have higher efficacy and overcome resistance coming from potential compensatory mechanisms; on the other hand, it will allow the downscaling of the doses of the drugs used, with a possible reduction of the associated toxic adverse effects. Notably, in a case report by Afzal and colleagues, a patient with metastatic UM treated with a combination of ipilimumab and nivolumab, following the progression with the single-agent, nivolumab, demonstrated a durable response without recurrence for more than 22 months after treatment (137).

## Conclusions

**Figure 1:**
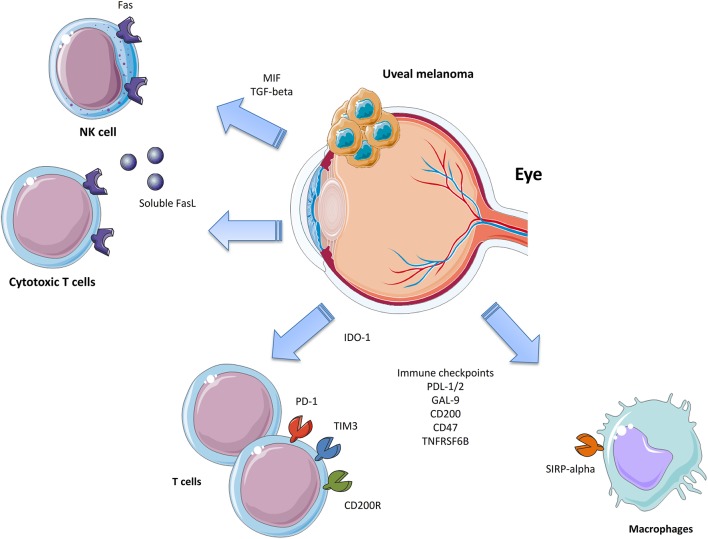
Immune-escape mechanisms in Uveal Melanoma.

Metastatic UM still represents an unmet medical need as there is no current approved treatment able to significantly increase the OS in patients. Chemotherapy has not proven successful and current immune-based therapies, despite the encouraging results coming from CM, have had unsatisfactory results. UM cells evade immune responses via several mechanisms that inhibit both the innate and adaptive immune system (Figure 1). It is therefore of the utmost importance to increase the understanding of the mechanisms put in place by UM to evade the immune surveillance in order to develop novel therapeutic strategies. It is likely that simultaneously targeting multiple immune-escape mechanisms may give an opportunity for the treatment of these patients. This, in fact, would allow them to overcome the unfavorable effects of boosting the immune responses, which in turn induce the establishment of additional immune-evading strategies, such as the upregulation of IDO-1, CD47, PD-L1, and MHC molecules. Promising results may be obtained, for instance, by the combination of TILs in association to anti-CD47 treatment or IDO-1 inhibition. Indeed, TILs-based therapies are currently ongoing and promising, but only partial responses are being observed. Monoclonal antibodies targeting CD47 are also under investigation in two Phase I trials on advanced solid and hematologic cancers (NCT02678338 and NCT02367196). The successful completion of these trials will provide more paths to follow in the search for novel and more effective management options for UM patients. Though not even preclinical proof of concept efficacy has so far been generated, with other “pathogenic”-tailored therapeutic approaches it can be expected that the emerging families of specific inhibitors of TGF-β (138) and MIF (139) also have the potential to be effective in some cases of UM.

## Author Contributions

MB, PF, FN, and MR designed and wrote the manuscript. EM, AL, AR, MF, VB, and TA reviewed and approved the final manuscript. All authors read and approved the final manuscript.

### Conflict of Interest

The authors declare that the research was conducted in the absence of any commercial or financial relationships that could be construed as a potential conflict of interest.
